# Imported severe *Plasmodium falciparum* infection in the first trimester of pregnancy complicated by post-artemisinin delayed hemolysis and intrauterine fetal death, a case report

**DOI:** 10.1186/s41182-023-00510-2

**Published:** 2023-05-12

**Authors:** Kohei Kamegai, Kayoko Hayakawa, Kei Yamamoto, Hidetoshi Nomoto, Kanako Komaki-Yasuda, Shigeyuki Kano, Norio Ohmagari

**Affiliations:** 1 Disease Control and Prevention Center, National Center for Global Health and Medicine, Tokyo, Japan; 2 Department of Tropical Medicine and Malaria, National Center for Global Health and Medicine, Tokyo, Japan

**Keywords:** *Plasmodium falciparum* malaria, Artemether-lumefantrine, Post-artemisinin delayed hemolysis, First-trimester, Blood transfusion

## Abstract

**Background:**

Post-artemisinin delayed hemolysis (PADH) is a serious complication in patients who recover from severe malaria after receiving artemisinin-based combined therapy (ACT), including artemether-lumefantrine. In Japan, among the antimalarial drugs recommended by the World Health Organization (WHO) guideline for severe malaria, intravenous quinine gluconate is available only in 29 designated hospitals, and intravenous artesunate is unavailable. Therefore, oral artemether-lumefantrine is occasionally administered as an alternative, even though it may be a suboptimal treatment. In non-endemic settings like Japan, a lack of knowledge of malaria and the side effects, such as post-artemisinin delayed hemolysis caused by the ACT, can have critical consequences. Like our patient, being a primigravida in the early stages of pregnancy is a serious risk factor for severe malaria and must be carefully monitored.

**Case presentation:**

This report describes a severe case of imported *Plasmodium falciparum* malaria complicated by fetal loss and prolonged anemia, requiring frequent blood transfusions. The patient was a previously healthy pregnant Japanese female in her 30 s. She developed a high fever 2 days after returning from Nigeria. The patient fulfilled the severe malaria criteria by WHO. On arrival, an abdominal ultrasound incidentally revealed a fetus of 5 week gestational age with a heartbeat in the uterus. Given her pregnancy and the severity of the disease, she was administered intravenous quinine 16 mg/kg as a loading dose. However, the second dose of quinine was not administered due to frequent vomiting and QTc prolongation. We initiated treatment with oral artemether-lumefantrine, and clearance of parasitemia was confirmed by microscopic observation on day 4. Miscarriage was noted on day 6 after admission. Moreover, the patient became feverish again up to 39 °C, and from days 14 to 22, the patient required multiple blood transfusions due to PADH. On day 40, follow-up was discontinued as the hemoglobin level exceeded 10 g/dL.

**Conclusions:**

In patients who recover from severe malaria after ACT treatment, monitoring the hemoglobin level for at least a month is strongly recommended for prompt identification of PADH. Travelers to malaria-endemic countries, especially primigravida women, should be provided with adequate information on the risk and prevention of infection.

## Background

In Japan, more than 50 imported malaria cases were reported annually before the COVID-19 pandemic, and of these, more than 60% were due to *Plasmodium falciparum* malaria [1].

Intravenous Artesunate is strongly recommended as the first line of treatment for severe malaria [2, 3]. Guidelines also recommend it for women suffering from severe malaria in the first trimester of pregnancy, despite inadequate evidence. If intravenous Artesunate is unavailable, intravenous quinine and oral artemether-lumefantrine (AL) can be used as alternative or interim drugs. However, among these, only oral AL is officially available in Japan. In fact, intravenous quinine gluconate is available only in the 29 designated hospitals for clinical studies [4], and intravenous artesunate is unavailable.

Post-artemisinin delayed hemolysis (PADH) is a severe complication that may occur following recovery from malaria after artemisinin-based combined therapy (ACT) [5, 6]. Although the incidence of PADH may be higher among non-immune individuals than in endemic areas, this side effect does not seem well-recognized in Japan. Herein, we describe a case of severe falciparum malaria with multiple complications and discuss the potential hazards caused by gaps in the knowledge of this condition.

## Case presentation

A pregnant Japanese woman in her late 30 s was referred to our infectious disease clinic in Tokyo for further management. Her chief complaint was fever for 4 days. Two days before the presentation, the patient sought medical attention at a local clinic near her home but could not obtain an appropriate diagnosis. She reported having returned to Japan 2 days before the onset of her fever after a 2-month stay in Nigeria. She also reported having experienced multiple mosquito bites during 2 weeks she spent there. She denied using malaria chemoprophylaxis, insect repellent, or mosquito nets. At the time of presentation at our clinic, her body temperature was 40.2 ℃, and her heart rate was 112/min; other vital signs were normal. She was alert, though mildly agitated and unable to sit due to weakness. Generalized jaundice was noted. On auscultation, both lungs were clear, and the chest X-ray was normal. Slight vaginal bleeding was also noted.

Screening tests for the human immunodeficiency virus and blood cultures were negative. Blood examination revealed hyperbilirubinemia, severe thrombocytopenia, elevated liver enzymes, and elevated d-dimer (Table 1). The arterial blood gas test did not reveal acidosis or hypoglycemia.

**Table 1:** Laboratory examination results on admission

*Blood*		*Urine*	
T-Bil	8.3 (mg/dL)	pH	6.0
D-Bil	5.4 (mg/dL)	Specific gravity	1.021
AST	94 (U/L)	Protein	1 +
ALT	149 (U/L)	Sugar	–
LDH	550 (U/L)	Ketone	**–**
CK	250 (U/L)	Occult blood	2+
BUN	32 (mg/dL)	Bilirubin	2+
Cre	1.04 (mg/dL)	Red blood cell	–
Na	128 (mEq/L)	White blood cell	–
K	3.6 (mEq/L)	*Venous blood gas*	
Cl	94 (mEq/L)	pH	7.362
Blood glucose	67 (mg/dL)	pCO2	42.3 (mmHg)
CRP	15.37 (mg/dL)	HCO3-	24.0 (mmol/L)
WBC	10,000 (/μL)	Lac	5.0 (mmol/L)
Hb	13.1 (g/dL)	AnionGap	7.8 (mmol/L)
Hct	35.2 (%)		
MCV	83.2 (fl)		
Platelets	1.8 (10^4^/μL)		
PT-INR	1.20		
APTT	36 (sec)		
Fib	136 (mg/dL)		
ATIII	59 (%)		
FDP	76.2 (μg/mL)		
D-dimer	46.7 (μg/mL)		

Urine human chorionic gonadotropin test was positive, and transvaginal ultrasound (TVU) revealed a gestational sac (GS) with a fetal heartbeat (FHB) equivalent to 5 week gestation. She had no history of pregnancy.

**Fig. 1:**
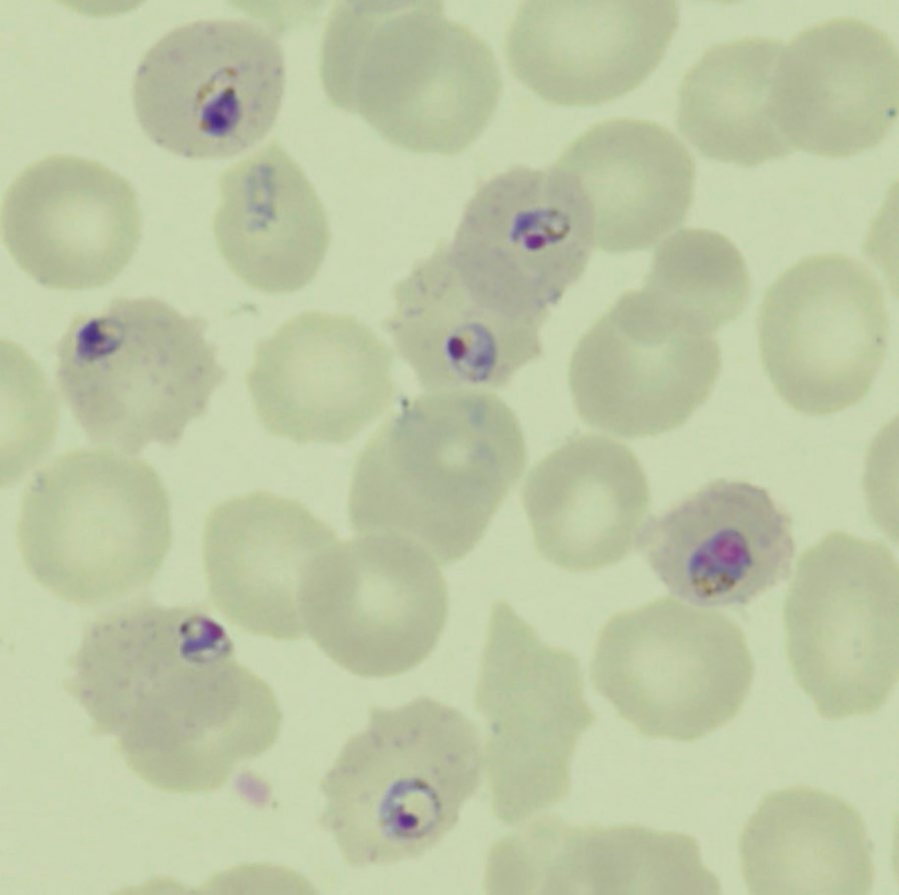
Blood smear on arrival showed *Plasmodium falciparum* trophozoites

Microscopic observation of the Giemsa-stained thin blood smear prepared from the patient's blood revealed *P. falciparum*-infected red blood cells at 7.3% parasitemia (Fig. 1), and the polymerase chain reaction methodalso verified *P. falciparum* infection. Thus,the diagnosis of P. *falciparum* malaria was made. The patient demonstrated two criteria of severe falciparum malaria as defined by the WHO: hyperbilirubinemia and prostration [2]. Soon after admission, 16 mg/kg of intravenous quinine gluconate was administered after obtaining informed consent to participate in the study "Efficacy and safety of injectable quinine in malaria patients (injectable quinine for malaria)" by the Development of Optimal Medical Care Network for the Diagnosis and Treatment of Tropical and Parasitic Diseases in Japan. However, due to frequent vomiting, hypoglycemia, and QTc prolongation, quinine was changed to a 3-day course of the oral AL (artemether 160 mg/day, lumefantrine 960 mg/day in total) on the following day. Serum lactate dehydrogenase (LDH), as a marker of hemolysis, reached the first peak (2389 IU/L) on day 3, then diminished.

The parasitemia disappeared by day 4 after starting treatment, but the temperature remained above 37.5 ℃ until day 16. Two sets of blood cultures retaken on day 14 were negative. She required two units (280 ml) of red blood cell concentrate on days 14 and 17, respectively. On day 17, she left the hospital at her request. After discharge, she continued monitoring her hemoglobin level at our outpatient clinic, and blood transfusions were still needed on days 19 and 21. Direct and indirect Coombs tests were negative on day 19, and haptoglobin was below the detection limit (< 10 mg/dL.) LDH reached the second peak (1226 IU/L) on day 21. On day 40, hemoglobin follow-up was discontinued as the hemoglobin level exceeded 10 g/dL.

**Fig. 2:**
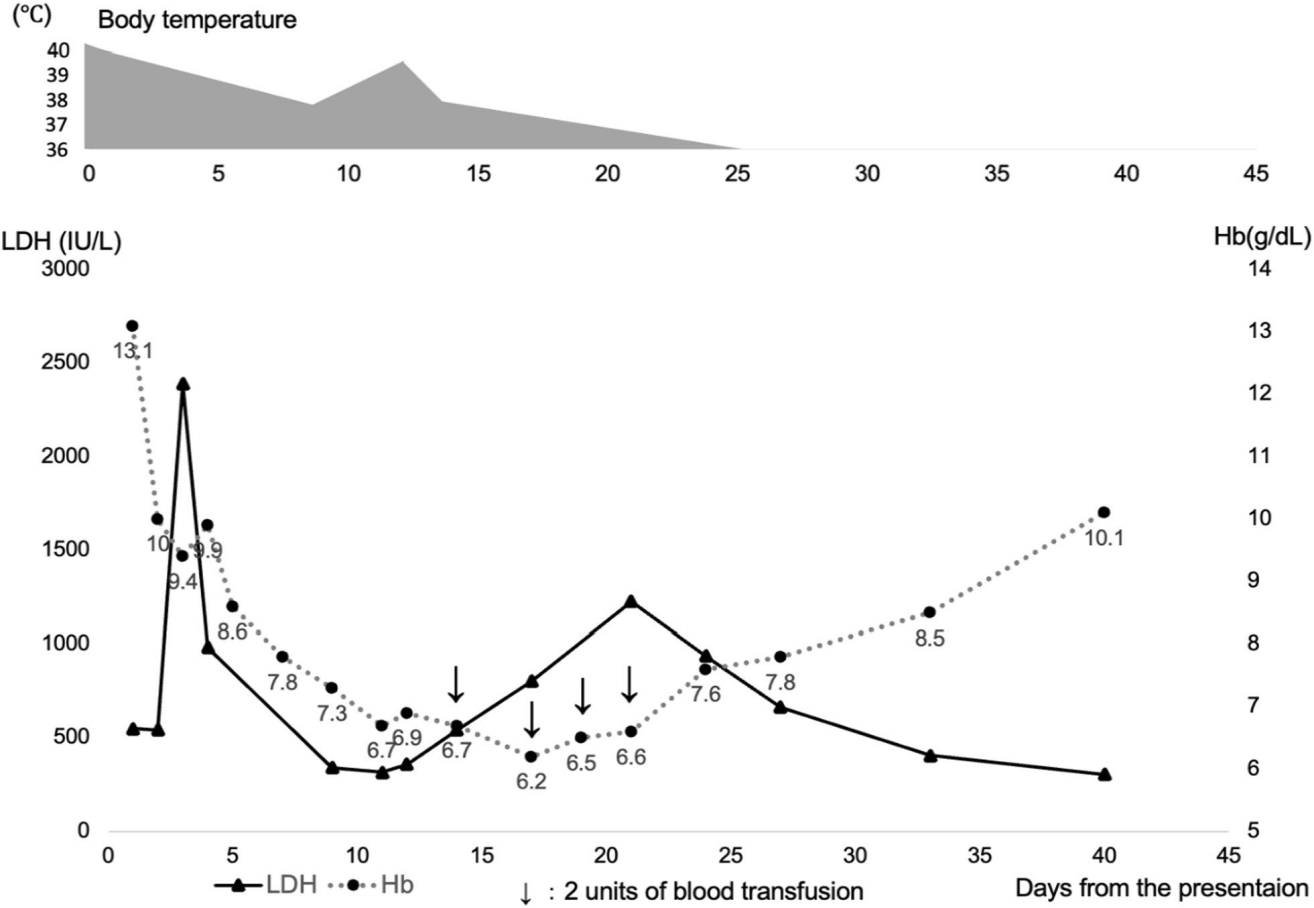
Clinical course of the patient. *LDH* lactate dehydrogenase, *Hb* hemoglobin. Downward arrows (↓) indicate administration of 2 units of blood transfusion

TVU on days 6 and 11 revealed a deformed GS without FHB, and subsequently, a diagnosis of miscarriage was made. On day 46, manual intrauterine aspiration was performed to evacuate uterine contents (Fig. 2).

## Conclusions and discussion

We have reported a case of imported falciparum malaria complicated with fetal loss and persistent transfusion-dependent anemia. PADH is a life-threatening condition seen 2–6 weeks after the ACT initiation in 20–30% of non-immune malaria patients, around 60% of whom require blood transfusions. PADH is defined as follows; a 10% decrease in hemoglobin associated with haptoglobin < 0.1 g/L and either an increase in LDH to > 390 IU/L or a > 10% rise 7 days after treatment initiation with the ACT [5, 6]. Like our case, hyperparasitemia is a major risk factor for PADH [5]. Treating severe PADH often requires blood transfusions. AL was the most probable cause of prolonged severe anemia in this case. Delayed hemolysis is rare in patients treated only with quinine [7]. Another differential diagnosis for hemolysis is black water fever (BWF.) BWF is associated with quinine use and G6PD deficiency (G6PDd) As we did not test G6PDd, BWF could not be sheerly excluded. However, the lack of hemoglobinuria and low prevalence of G6PDd made the possibility of BWF low. Systemic lupus erythematosus (SLE) can also explain both hemolysis and stillbirth in this case. However, we did not test SLE-associated antibodies, such as anti-double-stranded DNA antibody, anti-phospholipid antibody, and anti-nuclear antibody.

The possible pathophysiology for artemisinin-induced hemolysis is the shorter lifespan of erythrocytes infected with falciparum malaria compared with uninfected erythrocytes, known as the "pitting phenomenon" [8]. However, the etiology of prolonged hemolysis per se has yet to be explained. Bartoli et al*.* suggested the involvement of direct anti-globulin, but the literature review did not show a clinical difference between direct anti-globulin test-positive and negative patients [9]. A French group suggested falciparum HRP2-positivity using a dilution of whole blood of ACT-treated patients to predict the development of PADH with acceptable sensitivity and specificity [10].

To assess the risk of PADH following AL therapy, we searched articles in the English literature in PubMed describing oral AL-associated PADH following severe malaria. We excluded the cases relating to black water fever or uncomplicated malaria (Table 2) and found 12 cases, all of which reported falciparum malaria with marked parasitemia [11–19]. One of these was a fatal case [19]; however, it might be an outlier, because the patient refused blood transfusions due to religious beliefs. In Japan, blood transfusion is generally indicated for hemoglobin below 7 g/dL. In 10 out of 12 cases from our literature search, the nadir of the hemoglobin level was below 7 g/dL, and 5 of them received blood transfusions. This figure is far higher than a prospective study conducted on 27 patients with uncomplicated falciparum malaria [6]. In addition, in all the cases that describe the parasitemia clearance time, the hemoglobin level nadir came after the parasitemia clearance. This literature review strongly indicates that non-immune individuals are at a higher risk of developing PADH. Physicians should inform patients of the risk of PADH before prescribing ACT including AL. In addition, physicians must be reminded of the significance of continuing hemoglobin monitoring after the clearance of parasitemia at least once a week for a month after completion of therapy with AL.

**Table 2:** List of reported cases of severe malaria with PADH following oral arthemeter-lumefantrine therapy

Author	Age/sex	Ethnicity	Malaria type	Infection site	Previous malaria	Regimen	Parasitemia (% or/μL)	Peak LDH (IU/L)	Parasitemia clearance	Hb nadir (g/dL)	Blood transfusion	Coombs (direct/indirect)	Corticosteroid use	Result	Recurrence of PADH
Corpolongo et al*. *(2012) [11]	34 M	Italian	Pf	Africa	No	IVQ + DOXY → AL	6	2071	Yes (72 h)	5.9 (day 17)	Yes	Neg/Neg	No	Recovery	No
De Nardo et al*. *(2013) [12]	55 M	ND	Pf	Africa	Yes (30yrs ago)	AL	6	1326	Yes (48 h)	6.9 (day 8,22)	Yes	Pos/Neg	Yes	Recovery	No
Raffray et al. (2014) [13]	17 F	Ivoirian	Pf	Africa	No	AL → IVAS	0.8	658	Yes (< 192 h)	4.6 (day 14)	No	Pos/ND	Yes	Recovery	No
Tsuchido et al. (2017) [14]	20 M	Japanese	Pf	Africa	No	AL	20.6 (865,000)	1855	Yes (96 h)	7.2 (day 16)	No	Neg/Neg	No	Recovery	No
	27 F	Japanese	Pf	Africa	No	QH + DOXY → IVQG → AL	7.9	712	Yes (72 h)*	8.8 (day 11)	No	Neg/ND	No	Recovery	No
Lebrun et al. (2017) [15]	43 M	Caucasian	Pf	Africa	Yes (22yrs ago)	IVAS → AL	30	3035	Yes (72 h)	5.3 (day 18)	No	Pos/Neg	Yes	ND	No
Hasegawa et al*. *(2018) [16]	21 M	Japanese	Pf + Pv	India	No	AL → MQ → CQ + PQ	31.9 (920,000)	1400	Yes (48 h)	3.8 (day 9)	Yes	Neg/Neg	No	Recovery	No
Nakamura-Uchiyama et al*.* (2018) [17]	41 M	Nigerian	Pf	Africa	ND	AL	(672,000)	ND	Yes (48 h)	6.3 (day 19)	No	ND/ND	ND	ND	ND
	39 M	Japanese	Pf	Africa	ND	AL	(549,000)	ND	Yes (192–336 h)	6.5 (day 18)	No	ND/ND	ND	ND	ND
Conlon et al. (2020) [18]	24 M	ND	Pf	Africa	No	IVQD + DOXY → AL	12	1759	Yes (> 96 h)*	< 7	Yes	Pos/ND	No	Recovery	ND
Gustafsson et al*. *(2021) [19]	60 F	ND	Pf	Africa	No	AL	ND	925	ND	< 4.3	No†	Pos/ND	Yes	Death	−
Kamegai et al. (2023)	30sF	Japanese	Pf	Africa	No	IVQG → AL	7.3	2394	Yes (96 h)	6.2 (day 17)	Yes	Neg/Neg	No	Recovery	No

ND, not described; Pf, Plasmodium falciparum; Pv, Plasmodium vivax; AL, oral artemether-lumefantrine; IVAS, intravenous artesunate; IMA, intramuscular artemether; QH, oral quinidine hydrochloride; DOXY, doxycycline; IVQG, intravenous quinine gluconate; IVQ, intravenous quinine; IVQD, intravenous quinidine; MQ, mefloquine; CQ, chloroquine; PQ, primaquine; DHA-PPQ, dihydroartemisinin-piperaquine

^*^Parasitemia was almost undetectable (< 0.1%); however, complete clearance has not been described

^†^The patient refused to receive a blood transfusion because of religious issues

Some literature also suggests using corticosteroids if hemolysis is associated with an autoimmune mechanism, but evidence for this is lacking. In our case, corticosteroids were not used, because both direct and indirect Coombs tests were negative. We conducted a literature review on corticosteroid use in such cases. This review revealed five patients had positive direct Coombs test results [12–19]. Among them, four received corticosteroid therapy [12, 13, 15, 19], and two recovered without blood transfusions [13, 15], though the hemoglobin level nadir was below 7 g/dL. In one case, direct Coombs test was positive, while it had been negative 5 days earlier, after which it became negative again [13]. In severe PADH, it may be rational to repeat the Coombs test. Further study is needed to establish a strategy for early identification and appropriate management of PADH.

The patient in this report also happened to be a primigravida in the first trimester. Pregnancy is a well-known risk factor for severe malaria, and being a primigravida may predispose an individual to more severe disease [2, 3], which remains unknown. Being a primigravida and early pregnancy stages are factors for elevated human chorionic gonadotropin (hCG) levels. An experiment using a culture medium showed that the high hCG level did not enhance the in vitro growth of *P. falciparum*. A possible explanation is that the genome sequence of *P. falciparum* found no receptor homologous to that of humans [20]. However, since hCG contributes to angiogenesis in the uterus in the early stage of pregnancy, it may augment the sequestration of trophozoite-stage *P. falciparum*, causing severe disease [21]. The pathological findings of the placenta were non-contributory, and the direct cause of the abortifacient remained unknown. *P. falciparum* proliferates in the placenta [21]. *P. falciparum*-infected red blood cells interact with placental tissue and induce activation of inflammatory cytokines, such as tissue necrosis factor-alpha (TNF-α), causing intervilliositis. Intervilliositis reduces megalin, which transports essential proteins to the placenta, and an amino acid transporter supplies amino acids to the trophic membrane [21]. These mechanisms result in the development of placental ischemia, which may lead to fetal death.

Until recently, ACT was regarded as responsible for miscarriage, because animal experiments showed potential teratogenic effects. The previous versions of the WHO guideline [22] suggested using quinine plus clindamycin for pregnant women during the first trimester with uncomplicated malaria. However, recent reports indicated that the ACT was less harmful to the fetus than expected. In a meta-analysis, Dellicour et al. suggested that ACT was not associated with an increased risk of miscarriage or stillbirth [23]. The latest WHO guideline issued a new recommendation for using ACT in the first trimester [2]. Thus, we assume that the fetal loss in our case was due to falciparum malaria itself, although the side effects of antimalarial drugs cannot be completely ruled out from the list of possible causes. In addition, a chromosomal test of the fetus was not conducted in this case, which could have eliminated chromosomal abnormalities as the cause of fetal death.

This case emphasizes the importance of ascertaining obstetric status while assessing the risk status of female malaria patients. Non-immune women of reproductive age should be more carefully advised of the dangers before travel. Moreover, it may be justified to imbue them with the importance of contraception in risky areas or check pregnancy status before traveling.

In conclusion, physicians must monitor hemoglobin levels daily until parasitemia is cleared and weekly for at least 4 weeks after the clearance, especially in cases of severe malaria with high parasitemia. In addition, as being a primigravida and early stage of pregnancy are risk factors for severe malaria, non-immune women should be well-educated about risks and precautions to be taken before traveling to endemic areas.

## Data Availability

The authors confirm that the data supporting the findings of this study are available within the article. Raw data that support the findings of this study are available from the corresponding author, upon reasonable request.

## Abbreviations

PADH: Post-artemisinin delayed hemolysis
ACT: Artemisinin-based combined therapy
WHO: World Health Organization
AL: Artemether-lumefantrine
TVU: Transvaginal ultrasound
GS: Gestational sac
FHB: Fetal heartbeat
LDH: Lactate dehydrogenase
hCG: Human chorionic gonadotropin
TNF-α: Tissue necrosis factor-alpha
